# Biomarkers of central and peripheral inflammation mediate the association between HIV and depressive symptoms

**DOI:** 10.1038/s41398-023-02489-0

**Published:** 2023-06-06

**Authors:** Arish Mudra Rakshasa-Loots, Nicholas Bakewell, David J. Sharp, Magnus Gisslén, Henrik Zetterberg, Jasmini Alagaratnam, Ferdinand W. N. M. Wit, Neeltje A. Kootstra, Alan Winston, Peter Reiss, Caroline A. Sabin, Jaime H. Vera, on behalf of the COmorBidity in Relation to AIDS (COBRA) cohort

**Affiliations:** 1 Edinburgh Neuroscience, School of Biomedical Sciences, The University of Edinburgh, Edinburgh, UK; 2 Department of Global Health and Infection, Brighton and Sussex Medical School, University of Sussex, Brighton, UK; 3 Institute for Global Health, University College London, London, UK; 4 Department of Brain Sciences, Imperial College London, London, UK; 5 Care Research & Technology Centre, UK Dementia Research Institute, London, UK; 6 Department of Infectious Diseases, Institute of Biomedicine, Sahlgrenska Academy at University of Gothenburg, Gothenburg, Sweden; 7 Department of Infectious Diseases, Sahlgrenska University Hospital, Region Västra Götaland, Gothenburg, Sweden; 8 Department of Psychiatry and Neurochemistry, Institute of Neuroscience and Physiology, the Sahlgrenska Academy at the University of Gothenburg, Mölndal, Sweden; 9 Clinical Neurochemistry Laboratory, Sahlgrenska University Hospital, Mölndal, Sweden; 10 Department of Neurodegenerative Disease, UCL Institute of Neurology, Queen Square, London, UK; 11 UK Dementia Research Institute at UCL, London, UK; 12 Hong Kong Center for Neurodegenerative Diseases, Clear Water Bay, Hong Kong, China; 13 Wisconsin Alzheimer’s Disease Research Center, University of Wisconsin School of Medicine and Public Health, University of Wisconsin-Madison, Madison, WI USA; 14 Department of Infectious Disease, Imperial College London, London, UK; 15 Department of Sexual Health and HIV, Chelsea & Westminster Hospital NHS Foundation Trust, London, UK; 16 Stichting HIV Monitoring, Amsterdam, The Netherlands; 17 Amsterdam Institute for Infection and Immunity, Amsterdam, The Netherlands; 18 Amsterdam UMC location University of Amsterdam, Global Health, Amsterdam, The Netherlands; 19 Amsterdam Institute for Global Health and Development, Amsterdam, The Netherlands; 20 Department of Experimental Immunology, Amsterdam University Medical Centers, University of Amsterdam, Amsterdam, The Netherlands

**Keywords:** Predictive markers, Depression

## Abstract

People living with HIV are at increased risk for depression, though the underlying mechanisms for this are unclear. In the general population, depression is associated with peripheral and central inflammation. Given this, and since HIV infection elicits inflammation, we hypothesised that peripheral and central inflammatory biomarkers would at least partly mediate the association between HIV and depressive symptoms. People living with HIV (*n* = 125) and without HIV (*n* = 79) from the COmorBidity in Relation to AIDS (COBRA) cohort were included in this study. Participants living with and without HIV had similar baseline characteristics. All participants living with HIV were on antiretroviral therapy and were virally suppressed. Plasma, CSF, and brain MR spectroscopy (MRS) biomarkers were measured. Using logistic regression models adjusted for sociodemographic factors, we found that participants with HIV were more likely to have Any Depressive Symptoms (Patient Health Questionnaire [PHQ-9] score >4) (odds ratio [95% confidence interval] 3.27 [1.46, 8.09]). We then sequentially adjusted the models for each biomarker separately to determine the mediating role of each biomarker, with a >10% reduction in OR considered as evidence of potential mediation. Of the biomarkers analysed, MIG (−15.0%) and TNF-α (−11.4%) in plasma and MIP1-α (−21.0%) and IL-6 (−18.0%) in CSF mediated the association between HIV and depressive symptoms in this sample. None of the other soluble or neuroimaging biomarkers substantially mediated this association. Our findings suggest that certain biomarkers of central and peripheral inflammation may at least partly mediate the relationship between HIV and depressive symptoms.

## Introduction

Mental health is a key priority in improving the overall quality of life for people living with HIV. Depression, in particular, is one of the most common psychiatric disorders in people living with HIV. Early studies estimated that the prevalence of depression amongst people living with HIV is twice as high as that in the general population [1], though heterogeneity in screening tools used in studies makes a rigorous meta-analysis elusive [2]. More recent evidence has shown that HIV infection is associated with an increased likelihood of depression [3]. This risk may be exacerbated by “premorbid” depression, i.e., high prevalence of depression even before seroconversion in disenfranchised populations which are disproportionately affected by HIV. Amongst people living with HIV, smoking, drug use, excess alcohol use, or a recent sexually transmitted infection are further associated with greater prevalence of depressive symptoms [4]. Prevalence rates of depression also tend to differ by factors such as gender, socioeconomic status, or geography. Notably, the risk of depression is higher amongst women living with HIV and amongst people in the Global South [5, 6]. These high rates of depression impact the quality of life and ability to access care for people living with HIV. However, we do not currently fully understand why people living with HIV face this elevated risk for depression. Therefore, it is critical to examine (neuro)biological and psychosocial mechanisms that may drive this increased risk for depression. Understanding these underlying mechanisms may enable the discovery of predictive biomarkers as well as potential therapeutic targets for scalable interventions [7].

In the general population, depression is increasingly being recognised as comprising multiple distinct subtypes—constellations of behavioural disturbances, genetic risk factors, and neurobiological deficits—rather than a monolithic condition [8]. One such subtype of depression is associated with inflammation in the periphery and the central nervous system (CNS) [9]. Several sickness behaviours, such as fatigue, reduced appetite, and loss of interest or pleasure (motivational anhedonia), are shared across depression and inflammatory conditions [10]. People who experience depression exhibit increased concentrations of circulating pro-inflammatory cytokines and chemokines [11, 12]. Administration of anti-inflammatory medication as monotherapy or in conjunction with other antidepressant treatments can alleviate depressive symptoms [13]. Taken together, this evidence indicates that inflammation may play a role in the pathogenesis of depression, or at least a subtype of depression characterised by sickness behaviours. Although the precise relationship between neuroinflammation and depressive symptoms remains unclear, it is possible that neuroinflammatory responses contribute to neurotransmitter dysfunction, hypothalamus-pituitary-adrenal (HPA) axis dysregulation, and impaired hippocampal neurogenesis to generate features that are common to depression and sickness [14].

An inflammation-mediated subtype of depression may have important implications for our understanding of HIV-associated depression. The brain is a key reservoir for HIV infection, including for people living with HIV who are virally suppressed on antiretroviral therapy. HIV viral proteins and viral DNA elicit a neuroinflammatory response in the brain, characterised by sustained microglial activation [15]. Furthermore, systemic inflammation persists in spite of suppressive antiretroviral therapy [16, 17]. Given this, it is possible that HIV-induced peripheral and central inflammation may contribute to the increased risk for depression amongst people living with HIV and represent a promising screening and therapeutic target.

This study leveraged data from a well-characterised cohort of people living with and without HIV with similar demographic characteristics to evaluate the contributions of biomarkers of both central and peripheral inflammation to the association between HIV and depressive symptoms. We hypothesised that peripheral and central inflammatory biomarkers would at least partly mediate the association between HIV status and depressive symptoms.

## Methods

### Cohort

The COmorBidity in Relation to AIDS (COBRA) study was a prospective cohort of adults living with HIV in London (UK) and Amsterdam (Netherlands) recruited between 2013 and 2014 [18]. At enrolment, participants living with HIV in the cohort were aged 45 or over, virally suppressed, and on ART for at least 12 months. The cohort also included demographically-similar participants without HIV. All participants underwent a comprehensive battery of cognitive testing, blood and cerebrospinal fluid (CSF) sample collection for biomarker measurement, and neuroimaging at a baseline visit and a follow-up visit separated by 2 years [19]. This study was approved by the institutional review board of the Academic Medical Center (AMC) of the University of Amsterdam (reference number NL 30802.018.09) and a UK Research Ethics Committee (REC) (reference number 13/LO/0584 Stanmore, London). The current analysis included baseline data collected as part of the COBRA study.

### Patient Health Questionnaire

Depressive symptoms were measured using the nine-item Patient Health Questionnaire (PHQ-9). The PHQ-9 is a well-validated questionnaire that assesses self-reported frequency of depressive symptoms over the preceding two weeks [20]. The questionnaire is scored between 0 and 27, with a score of 10 commonly used as a cut-off to screen for major depressive disorder (MDD) [21]. All potential participants for the COBRA study previously completed a self-administered PHQ-9 questionnaire as part of the study screening. Individual items and total scores were recorded for each participant. Participants whose total score on the PHQ-9 at a screening visit was higher than 15 were ineligible for the COBRA cohort. For the current analysis, PHQ-9 score was summarised as a dichotomous variable (PHQ-9 score >4 classified as “Any Depressive Symptoms” as suggested by Kroenke et al. [20]) and as a continuous variable.

### Plasma biomarkers

Plasma concentrations of various biomarkers were previously determined for participants in the COBRA cohort, as described in detail elsewhere (Supplementary File S1 in [19]). Immunoturbidimetry was used to determine high-sensitivity CRP concentrations. Enzyme-linked immunosorbent assays (ELISAs) were used to determine the plasma concentrations of I-FABP, sCD14, sCD163, neopterin, and sCD16. A single molecular array (SIMOA) assay was used to measure plasma concentration of NFL. High-performance liquid chromatography was used to determine plasma concentrations of tryptophan and kynurenine, from which a kynurenine-to-tryptophan (Kyn:Trp) ratio was calculated.

An expanded set of pro-inflammatory cytokines and chemokines was previously measured only in a subset of 78 participants [19]. These 78 participants were randomly selected with equal numbers across COBRA age groups (45–50 years, 51–55 years, 56–60 years, 61–65 years, 66–70 years), except for the oldest age group (>70 years) where few individuals were available. For this subset, human magnetic Luminex assay was used to determine plasma concentrations of IL-6 and TNF-α (pro-inflammatory cytokines), and MIG/CXCL9, IP-10/CXCL10, MCP-1/CCL2, MIP1α/CCL3, and RANTES/CCL5 (chemokines).

### CSF biomarkers

Concentrations of biomarkers were likewise previously measured in cerebrospinal fluid (CSF), as described in detail elsewhere (Supplementary File S1 in [19]). ELISAs were used to determine the CSF concentrations of sCD14, sCD163, neopterin, and NFL. Liquid chromatography was used to determine the CSF Kyn:Trp ratio. As with plasma biomarkers, in a subset of 78 participants, Luminex assay was used to determine CSF concentrations of IL-6, TNF-α, MIG/CXCL9, IP-10/CXCL10, MCP-1/CCL2, MIP1α/CCL3, and RANTES/CCL5.

### Magnetic resonance spectroscopy

Proton magnetic resonance spectroscopy (^1^H-MRS, or MRS) data was acquired to assess concentrations of neurometabolites. Parameters of neuroimaging acquisition have been reported in detail elsewhere (Supplementary File S2 in [19]). For image acquisition during baseline COBRA study visits, a Siemens 3T Verio scanner was used at the London site, while Philips 3T Intera and 3T Ingenia scanners were used at the Amsterdam site. Single-voxel MRS data were acquired for two regions: frontal white matter (FWM) and putamen. Concentrations of choline [Cho] and myo-inositol [mI] were determined for these regions [22].

A summary of biomarkers included in this study, along with the number of participants for whom each biomarker was assessed, is provided in Table 1.

**Table 1:** Summary of neuroimaging, plasma, and cerebrospinal fluid (CSF) biomarkers of (neuro)inflammation included in the study.

		Marker of	FWM	*N*	Putamen	*N*
Neurometabolites	Myo-inositol	Glial cell activation	x	161	x	63
	Choline	Glial cell activation	x	184	x	128

		Marker of	Plasma	*N*	CSF	*N*
Soluble biomarkers measured in all participants, where possible	CRP	Acute phase response	x	204	–	–
	I-FABP	Intestinal barrier integrity	x	202	–	–
	Kyn:Trp Index	IDO-1 activity	x	203	x	202
	Neopterin	Inflammation	x	203	x	202
	NFL	Neuronal injury	x	202	x	203
	sCD14	Monocyte activation	x	201	x	202
	sCD16	Monocyte activation	x	202	–	–
	sCD163	Monocyte activation	x	204	x	203

		Marker of	Plasma	*N*	CSF	*N*
Soluble biomarkers measured in a subset of participants	IL-6	Inflammation	x	78	x	78
	IP-10/CXCL10	Chemotaxis	x	78	x	78
	MCP-1/CCL2	Chemotaxis	x	78	x	78
	MIG/CXCL9	Chemotaxis	x	78	x	78
	MIP1α/CCL3	Chemotaxis	x	78	x	78
	RANTES/CCL5	Chemotaxis	x	78	x	78
	TNF-α	Inflammation	x	78	x	78

*FWM* frontal white matter.

Some soluble biomarkers were measured in all participants, where possible. An expanded set of pro-inflammatory cytokines and chemokines was measured only in a subset of *n* = 78 participants. The number of participants for whom data were available for each biomarker is indicated. ‘x’ indicates that a biomarker was measured in the respective brain region or biofluid, whereas ‘–’ indicates that it was not.

### Statistical analysis

We summarised participant characteristics using counts (proportions) for categorical variables and medians (interquartile ranges [IQRs]) for continuous variables. Univariate comparisons by HIV status were conducted using Wilcoxon-rank sum, Chi-squared (with Yates correction), Cochran-Armitage trend, and Fisher’s exact tests, as appropriate.

Following the assumptions of previous COBRA analyses, participants with biomarker values out of range were assumed to have a biomarker value of half the detection limit (e.g., if the detection limit was 0.30, then a value of 0.15 was assumed). The number of participants for whom this assumption was made for each biomarker is shown in Supplementary File 1. For biomarkers with a lower limit of 0 (namely high-sensitivity CRP), zero values were set to 0.01 so that the value could be log_2_-transformed (note that this only affected one participant in the dataset).

To assess correlations between concentrations of biomarkers measured in this sample, we determined Spearman’s rank correlation coefficients (*ρ*) using pairwise complete observations. We calculated correlations between all possible biomarker pairs, and additionally for subsets of plasma-plasma, CSF-CSF, plasma-CSF, and MRS biomarkers. Plasma-CSF correlations were calculated for the full sample, and for subgroups of participants by HIV status and depressive symptom status.

To assess the main effect of HIV status on the prevalence of “Any Depressive Symptoms”, we used logistic regression models to estimate the odds ratio (OR) (95% confidence interval [CI]) for the association between HIV status and Any Depressive Symptoms for our full sample with outcome data available (*N* = 204), with the reference OR = 1.00 for participants without HIV. To assess the main effect of HIV status on each biomarker, we used linear regression models for the outcome log_2_-transformed biomarker concentration, fitted separately for each biomarker and adjusted for sociodemographic factors (age, sex, ethnicity, and years of education).

For our primary analyses, we used logistic regression to explore the potential mediating role of biomarkers on the association between HIV status and “Any Depressive Symptoms”. We first adjusted the model for sociodemographic factors: age (years, continuous), sex, ethnicity, and years of education (continuous). Models involving any neurometabolite measures were further corrected for MRI scanner. The sample size considered for each model is reported, as this varied based on the availability of data for each biomarker of interest. To determine whether any biomarkers mediated the relationship between HIV status and Any Depressive Symptoms, we then sequentially adjusted the model for each (log_2_-transformed) biomarker separately. We report the OR for the association between HIV status and Any Depressive Symptoms for all models, along with profile-likelihood CIs. Individual biomarkers for which adjustment resulted in a >10% reduction in the OR were considered potential mediators of the association between HIV status and Any Depressive Symptoms. A reduction (rather than an increase, which may more likely reflect confounding) in the effect estimate after adjustment is commonly used to identify potential mediator variables [23].

For our sensitivity analyses, we used linear regression to explore the main effect of HIV status on depressive symptom severity, with PHQ-9 score as a continuous outcome. The same sequential adjustments for sociodemographic factors and individual biomarkers (with each biomarker tested in a separate model) were made for the sensitivity analyses as described for the primary analyses.

All analyses were conducted using listwise deletion, only including participants with data available on all variables used in an analysis. Analyses were performed using R version 4.1.0. A detailed description of assumptions made as part of these analyses is available in Supplementary File 2. We did not adjust for multiple testing, as we focus on the strengths of observed associations and consistency of results with sensitivity analyses, rather than on statistical significance.

## Results

### Participant characteristics

We included *N* = 204 participants for whom data were available on PHQ-9 score and at least one biomarker. Of these, *n* = 125 were participants living with HIV and *n* = 79 were demographically comparable controls. Sociodemographic, lifestyle, and HIV-specific characteristics, as well as the PHQ-9 responses, are summarised in Table 2. The median age [IQR] of participants was 57 [51–62] years, 92.6% of participants were male, 91.7% were White, and 82.8% were men who have sex with men. The median [IQR] years of education was 15 [13**–**16] years. All participants living with HIV were on combination antiretroviral therapy (cART) and had plasma HIV-RNA < 200 copies/mL.

**Table 2:** Summary of selected baseline characteristics and outcome data (PHQ-9 score/categories) by HIV status for participants from the COBRA cohort included in the current study.

Variable *n* (%) or median (interquartile range)	Overall *N* = 204	Participants without HIV *N* = 79	Participants with HIV *N* = 125	*p* value
*General baseline characteristics*				
Study Site				0.66
Amsterdam	124 (60.8%)	50 (63.3%)	74 (59.2%)	
London	80 (39.2%)	29 (36.7%)	51 (40.8%)	
Age (years)	57 (51, 62)	57 (52, 64)	55 (51, 62)	0.25
Sex at birth				>0.99
Female	15 (7.4%)	6 (7.6%)	9 (7.2%)	
Male	189 (92.6%)	73 (92.4%)	116 (92.8%)	
Men who have sex with men	169 (82.8%)	62 (78.5%)	107 (85.6%)	0.26
Ethnicity				0.03
Black-African	17 (8.3%)	2 (2.5%)	15 (12.0%)	
White	187 (91.7%)	77 (97.5%)	110 (88.0%)	
Years of Education	15 (13, 16)	16 (14, 16)	14 (13, 16)	0.19
Current smoker	58 (28.4%)	20 (25.3%)	38 (30.4%)	0.53
Current alcohol use	173 (84.8%)	73 (92.4%)	100 (80.0%)	0.03
Use of recreational drugs in the past 6 months	59 (28.9%)	18 (22.8%)	41 (32.8%)	0.17
Ever injected drugs	5 (2.5%)	0 (0.0%)	5 (4.0%)	0.16
*HIV-specific characteristics*				
Years since HIV diagnosis			14.8 (9.0, 18.7)	
Currently on any form of combination antiretroviral therapy (cART)			125 (100.0%)	
Duration of cART (years)			11.9 (7.3, 15.5)	
HIV-RNA viral load <200 copies/mL			125 (100.0%)	
Prior AIDS event			40 (32.0%)	
Current CD4 + T-cell count (cells/µL)			615 (472, 798)	
Nadir CD4 + T-cell count (cells/µL)			180 (100, 250)	
*PHQ-9 score*				
Total Score	2 (0, 4)	1 (0, 3)	2 (1, 5)	0.005
PHQ-9 > 4 (“Any Depressive Symptoms”)	42 (20.6%)	9 (11.4%)	33 (26.4%)	0.02

Univariate comparisons by HIV status were conducted using Wilcoxon-rank sum, Chi-squared (with Yates correction), Cochran-Armitage and Fisher’s exact tests, as appropriate.

Participants living with and without HIV were similar on most baseline sociodemographic and lifestyle characteristics, with notable exceptions: participants living with HIV were less likely to be White (88.1% vs 97.5%, *p* = 0.03) and current alcohol users (77.6% vs 92.4%, *p* = 0.03) compared to participants without HIV.

### Correlations between CSF and plasma inflammatory biomarkers

**Fig. 1:**
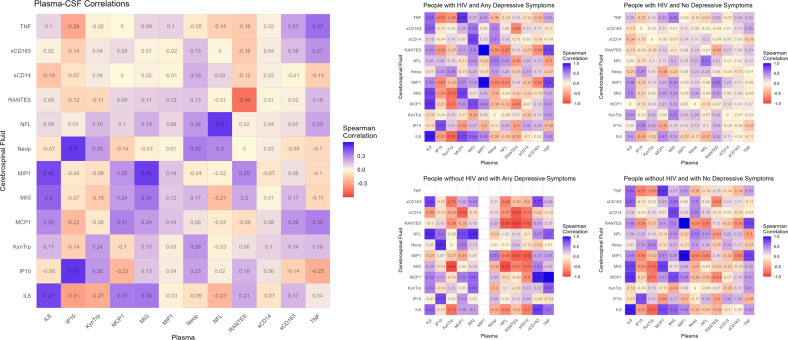
Correlations between cerebrospinal fluid (CSF) and plasma concentrations of soluble biomarkers measured in the current study. Correlations are shown for the full sample, and for subgroups of participants: people with HIV and Any Depressive Symptoms (maximum sample *n* = 33), people with HIV and No Depressive Symptoms (maximum sample *n* = 92), people without HIV and with Any Depressive Symptoms (maximum sample *n* = 9), and people without HIV and with No Depressive Symptoms (maximum sample *n* = 70). For correlations in the full sample, the strength of correlations are graded across a 3-point scale selected to range from the 5th and 95th percentiles of the correlation coefficients to optimise visual comparisons: −0.6 (in deep red), 0.0 (in light yellow), and +0.6 (in deep blue). For correlations in subgroups, the grading scale was: −1.0 (in deep red), 0.0 (in light yellow), and +1.0 (in deep blue).

The correlation matrix for soluble biomarkers which were measured in both plasma and CSF is shown in Fig. 1. Correlations are also reported for subgroups: people with HIV and Any Depressive Symptoms (maximum sample *n* = 33), people with HIV and No Depressive Symptoms (maximum sample *n* = 92), people without HIV and with Any Depressive Symptoms (maximum sample *n* = 9), and people without HIV and with No Depressive Symptoms (maximum sample *n* = 70). Note that, given smaller sample sizes in subgroups, certain biomarkers had identical values for all participants, resulting in an incalculable correlation coefficient due to lack of variability (i.e., the standard deviation of 0). Further matrices for correlations between imaging biomarkers only, plasma-plasma biomarkers only, CSF-CSF biomarkers only, and all soluble biomarkers are available in Supplementary File 3.

Overall, concentrations of soluble biomarkers were only weakly or moderately correlated, if at all (mean [*M*] = 0.07, standard deviation [SD] = 0.17, range −0.46 to 0.62). Moderate-to-strong positive correlations were observed between plasma Kyn:Trp ratio and plasma neopterin ($\hat \rho$ = 0.62), CSF IP-10 and CSF neopterin ($\hat \rho$ = 0.58), and CSF sCD14 and CSF sCD163 ($\hat \rho$ = 0.58). Notably, a cluster of moderate positive correlations was observed for plasma IL-6 with MIP1-α (CSF, $\hat \rho$ = 0.46), MCP-1 (plasma, $\hat \rho$ = 0.40), and MIG (plasma, $\hat \rho$ = 0.46; CSF, $\hat \rho$ = 0.40), and for CSF IL-6 with MIP1-α (CSF, $\hat \rho$ = 0.53), MCP-1 (CSF, $\hat \rho$ = 0.50), and MIG (CSF, $\hat \rho$ = 0.47).

Correlations between plasma and CSF concentrations of a single biomarker, represented by the diagonal in Fig. 1, tended to be slightly higher than the overall correlation of soluble biomarkers (*M* = 0.24, SD = 0.27, range = 0.46 to 0.51). The strongest correlations observed, which were still only moderate, were positive correlations between plasma and CSF IP-10 ($\hat \rho$ = 0.51), NFL ($\hat \rho$ = 0.50), and IL-6 ($\hat \rho$ = 0.47) and a negative correlation between plasma and CSF RANTES ($\hat \rho$ = -0.46). There were no strong correlations between plasma and CSF concentrations of any other soluble biomarkers, or between the putamen and frontal white matter (FWM) measures of neurometabolites (Supplementary File 3).

### Association of HIV separately with depressive symptoms and inflammation

There was a relatively low severity of depressive symptoms across all participants in this sample, with a median [IQR] PHQ-9 score of 2 [0, 4]. Despite this, depressive symptom scores were higher amongst participants living with HIV compared to participants without HIV (*p* = 0.005), with the prevalence of “Any Depressive Symptoms” also being higher in this group (26.4% vs 11.4%, *p* = 0.02, Table 2). The full range of PHQ-9 scores was 0 to 22, with four participants living with HIV reporting a PHQ-9 score >15 at the study visit (but not at the screening visit).

In the full sample, the crude (unadjusted) OR (95% CI) for the association between HIV status and Any Depressive Symptoms was 2.79 (1.30, 6.54). After adjusting for age, sex, ethnicity, and years of education, the adjusted OR increased to 3.27 (1.46, 8.09).

We found associations between HIV status and plasma concentrations of CRP, I-FABP, Kyn:Trp ratio, neopterin, NFL, sCD14, sCD16, sCD163, and TNF-α, and between HIV status and CSF concentrations of IP-10, neopterin, and Kyn:Trp ratio, (all *p* < 0.05, Supplementary File 4). Mean concentrations of these biomarkers were all higher amongst participants living with HIV compared to those without HIV.

### Mediation by biomarkers of peripheral and central inflammation

**Fig. 2:**
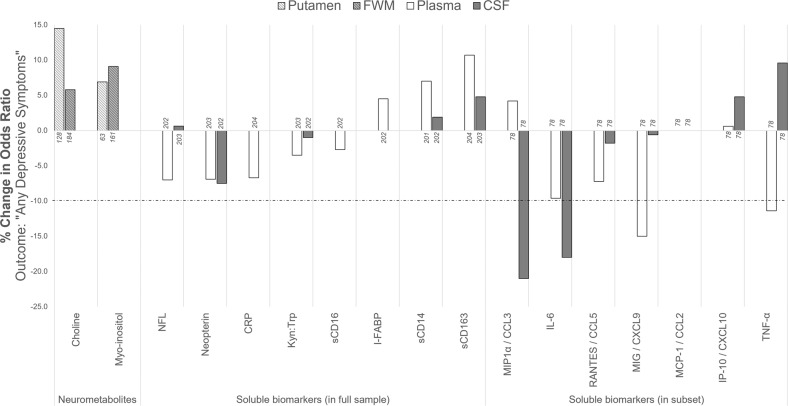
Mediation of the association between HIV status and Any Depressive Symptoms by adjusting for each biomarker separately. Sample sizes for which data were available for each biomarker are indicated at the *x* axis next to each bar. The dotted line marks a 10% reduction in the odds ratio, which represents our criterion for potential mediation.

The odds ratios for the association between HIV and Any Depressive Symptoms, before and after adjusting for each biomarker separately, are shown in Table 3. Of the biomarkers analysed, our criterion for potential mediation was met for plasma MIG (change in OR_HIV_: −15.0%), plasma TNF-α (−11.4%), CSF MIP1-α (−21.0%), and CSF IL-6 (−18.0%) (Fig. 2). None of the other plasma, CSF, or neuroimaging biomarkers met our criterion of greater than 10% reduction in OR.

**Table 3:** Odds ratios (OR, with 95% confidence interval [CI]) for the association between HIV status and Any Depressive Symptoms, before and after adjustment for each biomarker fitted separately.

Biomarker	*N*	*N* Any Depressive Symptoms	OR (95% CI) for Any Depressive Symptoms, adjusted for HIV status and sociodemographic factors	
			Before adjusting for biomarker	Adjusted for biomarker
*Neurometabolites*^a^				
*Myo*-inositol				
Frontal White Matter	161	29	2.96 (1.12, 9.13)	3.23 (1.19, 10.15)
Putamen	63	20	5.19 (1.32, 27.63)	5.55 (1.35, 30.35)
Choline				
Frontal White Matter	184	36	2.75 (1.17, 7.20)	2.91 (1.21, 7.74)
Putamen	128	29	7.46 (2.26, 35.53)	8.54 (2.50, 41.47)
*Soluble biomarkers measured* *in all participants, where possible*				
Plasma				
CRP	204	42	3.27 (1.46, 8.09)	3.05 (1.34, 7.61)
I-FABP	202	41	3.81 (1.65, 9.91)	3.98 (1.62, 10.86)
Kyn:Trp	203	41	3.76 (1.63, 9.78)	3.63 (1.54, 9.59)
Neopterin	203	41	3.76 (1.63, 9.78)	3.50 (1.44, 9.45)
NFL	202	41	3.16 (1.41, 7.80)	2.94 (1.29, 7.31)
sCD14	201	40	3.01 (1.34, 7.49)	3.22 (1.39, 8.20)
sCD16	202	42	3.36 (1.50, 8.30)	3.27 (1.44, 8.18)
sCD163	204	42	3.27 (1.46, 8.09)	3.62 (1.59, 9.11)
CSF				
Kyn:Trp	202	41	3.07 (1.37, 7.57)	3.04 (1.34, 7.62)
Neopterin	202	41	3.07 (1.37, 7.57)	2.84 (1.25, 7.11)
NFL	203	41	3.13 (1.40, 7.80)	3.15 (1.40, 7.82)
sCD14	202	41	3.08 (1.38, 7.62)	3.14 (1.39, 7.78)
sCD163	203	41	3.13 (1.40, 7.72)	3.28 (1.46, 8.13)
*Soluble biomarkers measured* *in a subset of 78 participants*				
Plasma				
IL-6	78	13	1.67 (0.46, 6.58)	1.51 (0.40, 6.01)
IP-10/CXCL10	78	13	1.67 (0.46, 6.58)	1.68 (0.45, 6.69)
MCP-1/CCL2	78	13	1.67 (0.46, 6.58)	1.67 (0.46, 6.59)
MIG/CXCL9	78	13	1.67 (0.46, 6.58)	1.42 (0.38, 5.63)
MIP1-α/CCL3	78	13	1.67 (0.46, 6.58)	1.74 (0.47, 7.00)
RANTES/CCL5	78	13	1.67 (0.46, 6.58)	1.55 (0.41, 6.20)
TNF-α	78	13	1.67 (0.46, 6.58)	1.48 (0.39, 5.98)
CSF				
IL-6	78	13	1.67 (0.46, 6.58)	1.37 (0.35, 5.59)
IP-10/CXCL10	78	13	1.67 (0.46, 6.58)	1.75 (0.46, 7.16)
MCP-1/CCL2	78	13	1.67 (0.46, 6.58)	1.67 (0.45, 6.58)
MIG/CXCL9	78	13	1.67 (0.46, 6.58)	1.66 (0.45, 6.58)
MIP1-α/CCL3	78	13	1.67 (0.46, 6.58)	1.32 (0.35, 5.26)
RANTES/CCL5	78	13	1.67 (0.46, 6.58)	1.64 (0.45, 6.47)
TNF-α	78	13	1.67 (0.46, 6.58)	1.83 (0.49, 7.31)

All models were adjusted for age, sex, ethnicity and years of education.

^a^Models which included neurometabolite measures were further corrected for scanner.

Conclusions from sensitivity analyses in which the PHQ-9 score was modelled as a continuous outcome were consistent with those of our main analyses (Supplementary File 5).

## Discussion

In this study, we aimed to determine whether biomarkers of peripheral and central inflammation may mediate the risk for depressive symptoms in HIV. We observed that the prevalence and severity of depressive symptoms were significantly greater amongst participants living with HIV than those without HIV, as were the concentrations of several plasma and CSF inflammatory biomarkers. These findings indicate that HIV status was associated both with increased risk for depressive symptoms and increased systemic and central nervous system inflammation. Crucially, we show that four biomarkers of inflammation—MIG and TNF-α in plasma, and MIP1-α and IL-6 in CSF—are potential mediators of the association between HIV status and depressive symptoms, as their inclusion in the regression models attenuated the odds ratio for this association. Taken together, our findings offer support for the hypothesis that the association between HIV and depressive symptoms may at least in part be mediated by biomarkers of central and peripheral inflammation.

Despite the biologically plausible conceptual links between neuroinflammation, depression, and HIV (described in detail elsewhere [24]), few studies have previously attempted to quantitatively test this hypothesis. Amongst participants living with HIV (but not those without HIV), Saloner et al. [25] observed a trend between depression severity and composite neuroinflammation scores while Woods et al. [26] similarly saw a significant association between specific dimensions of depression and plasma BDNF. In line with our findings in the current study, Musinguzi et al. [27] previously demonstrated that participants living with HIV recruited in Uganda who exhibited increased TNF-α were at significantly higher risk for depression. Our study offered the additional advantage of replicating this mediation effect in a sample of participants living with and without HIV. Given that TNF-α appears to mediate the relationship between HIV and depressive symptoms in two independent and demographically distinct samples, it is plausible that the effect of plasma TNF-α on this association is robust and reliable.

We found CSF IL-6 to have a considerable mediating role in depressive symptoms, which is compatible with previous evidence consistently implicating IL-6 in the risk for depression, although our study is one of the first to demonstrate this specifically in the context of HIV. We also saw sub-threshold but notable mediation of the association between HIV and depressive symptoms by plasma IL-6. The association between IL-6 and depression has been observed repeatedly with varying strengths [28]. Longitudinal twin studies and meta-analyses indicate that elevation in IL-6 may be a risk factor leading to depression, rather than a consequence of depression [29, 30]. Our findings therefore suggest that IL-6 may play a role in mediating the risk for depression in people living with HIV.

We found a moderate correlation between IL-6 concentrations in blood and CSF. We also observed moderate correlations for both plasma and CSF IL-6 with a cluster of chemokines, which suggests that the pro-inflammatory cytokine IL-6 may play an important role in chemotactic cascades in the periphery and the CNS. These results are compatible with findings from a previous study which similarly observed moderate correlations between blood and CSF IL-6 and between IL-6 and certain chemokines amongst people living with HIV [31]. Most other soluble biomarkers were only weakly or moderately correlated with each other in our sample. Correlations between plasma and CSF concentrations of individual biomarkers were slightly stronger on average, though still only moderate, which may be in part because participants living with HIV in this study were virally suppressed as measured by an undetectable plasma HIV-RNA load [32]. These findings may suggest that multiple inflammatory biomarkers may carry unique information about facets of the inflammatory cascade.

A novel and exciting finding in our exploratory study is the substantial mediation of the association between HIV and depressive symptoms by CSF MIP1-α. Although the functions of MIP1-α in chemotaxis and HIV suppression have been closely studied [33–35], few studies have attempted to understand what role, if any, this chemokine may play in depression. In fact, a recent meta-analysis investigating the role of chemokines in depression found only six eligible studies that tested associations between MIP1-α and depression [12]. This meta-analysis reported that blood MIP1-α concentrations were increased in depressed vs non-depressed individuals, but only when including participants who were physically healthy (i.e., without co-morbid physical illness). We observed a fairly large attenuation of the risk for depressive symptoms by CSF MIP1-α in a sample of participants living with and without HIV. Our findings thus suggest that MIP1-α may be a useful and underexplored biomarker for depression, especially in people living with HIV.

Notably, we did not find that the neuroimaging biomarkers measured in our study (mI and Cho in putamen and FWM) attenuated the odds ratio for the association between HIV status and depressive symptoms. These results indicate that alterations in concentrations of mI and Cho, which are often considered markers of glial cell activation and cellular metabolism [36], did not mediate the relationship between HIV and depressive symptoms in this sample. However, others have previously noted that changes in mI and Cho concentrations, as measured by MRS, may be caused by competing mechanisms and not specifically reflective of neuroinflammation [37, 38]. More sensitive techniques such as diffusion-weighted MRS, which has recently been shown to be sensitive to an experimental model of neuroinflammation in humans [39], may thus be used in future to clarify the relationship between imaging biomarkers of neuroinflammation and depressive symptoms in people living with HIV.

A few potential limitations must be considered in the interpretation of our findings. Our threshold for >10% reduction in OR for the association between HIV and depressive symptoms, which we used to define potential mediators in this study, may be considered low. Since this study was cross-sectional, we cannot make inferences about causality and cannot entirely rule out the possibility of other confounding variables which may lead to an artificial association of some of these biomarkers with the outcome. Additionally, the biomarkers included in this study were fixed by parent study design and chosen for their relevance to immune dysfunction, metabolism, and neuronal damage in age-associated non-communicable co-morbidities amongst people living with HIV. Some key biomarkers of inflammation and neurogenesis such as IL-1β and BDNF, which have been associated with depressive symptoms in people living with HIV, were not measured in this study but warrant investigation in future research [26, 40]. Concentrations of certain biomarkers such as MIP1-α in plasma and RANTES and TNF-α in CSF were near the detection limit for the majority of participants, which further limits these analyses.

Further limitations relate to the size and characteristics of the sample included in this study. The COBRA study protocol excluded participants with a total PHQ-9 score >15 at screening, thus these analyses were restricted to people without severe depressive symptoms, and most participants meeting criteria for “Any Depressive Symptoms” reported mild or moderate depressive symptom severity. Particularly for some biomarkers in this study, the sample of people with Any Depressive Symptoms was quite small, so some of these analyses may be underpowered. Given the small sample sizes in subgroups for correlation analyses (especially for participants without HIV and with Any Depressive Symptoms), limited meaningful comparisons between biomarker correlations in the full sample and in subgroups can be made. All participants with HIV were on antiretroviral medication, with potential side-effects such as fatigue which overlap with depressive symptoms, thus potentially confounding our observations. Participants needed to be willing to attend additional study visits and were encouraged to undergo lumbar puncture, which may further have introduced some selection bias. Finally, the demographic characteristics of this cohort were largely fixed by study design and were chosen to reflect the epidemiology of older adults living with HIV in the UK and the Netherlands at the time of recruitment (2013–14). As a result, this cohort was comprised primarily of White men who have sex with men, which is not representative of the global population of people living with HIV. This lack of gender and ethnic diversity in our sample limits generalisation to other populations, including women living with HIV and people living with HIV in the Global South.

Despite these limitations, our study offers novel and significant implications for our understanding of HIV-associated depression. Through this exploratory study, we offer evidence for the mediating role of pro-inflammatory cytokines (TNF-α, IL-6) and chemokines (MIP1-α, MIG) in the association between HIV and depressive symptoms. This is one of the first studies to specifically investigate the interrelationship between systemic and neuroinflammation, depressive symptoms, and HIV in a relatively large sample of participants living with HIV and carefully selected demographically-similar controls. We also report a rich dataset of correlations between plasma and CSF concentrations of a wide range of inflammatory biomarkers, which is often missing from similar studies, and which may inform future research design and biomarker discovery. This study particularly adds value to the ongoing efforts for the discovery of clinically useful biomarkers for depression, especially in the context of HIV, by revealing MIP1-α, an underexplored biomarker, as a notable mediator of the risk for HIV-associated depression. It will be important to replicate these findings in larger and more diverse cohorts and with participants who exhibit greater depressive symptom severity. We did not include interaction terms or stratify our analyses to explore any variable-specific trends due to the limited sample size and small number of participants with Any Depressive Symptoms, but future studies with larger samples may investigate age- and sex-specific trends. In addition, we did not explore possible synergistic or antagonistic effects of biomarkers in this study, but future studies may analyse the effects of multiple biomarkers in combination on the relationship between HIV and depressive symptoms. Future studies may also investigate the precise mechanisms by which the inflammatory biomarkers highlighted in our study influence the risk for depression, perhaps by exploring possible links between these biomarkers and microglial activation, synaptodendritic injury and neurogenesis, or serotonin depletion.

## Supplementary information

Supp File 1 - Undetectable Biomarkers

Supp File 2 - Statistical Analyses

Supp File 3 - Correlation Matrices

Supp File 4 - Biomarkers by HIV Status (Linear Regression)

Supp File 5 - Sensitivity Analyses (Linear Regression)

Supp Figure 1 - Imaging Correlations

Supp Figure 2 - CSF-CSF Correlations

Supp Figure 3 - Plasma-Plasma Correlations

Supplementary Figures

## Data Availability

The datasets presented in this article are not readily available. Data sharing has been restricted by the Medisch Ethische Toetsingscommissie and the UK National Research Ethics Service (NRES) because the data underlying this study contains sensitive and potentially identifying information. Requests for data sharing, however, can be made on a case-by-case basis following the submission of a concept sheet as per instructions on the project website (http://fp7-cobra.eu/). Once submitted, the proposed research/analysis will undergo review by the COBRA Steering Committee for evaluation of the scientific value, relevance to the study, design and feasibility, statistical power and overlap with existing projects. If the proposed analysis is for verification/replication, data will then be made available. If the proposed research is for novel science, upon completion of the review, feedback will be provided to the proposer(s). In some circumstances, a revision of the concept may be requested. If the concept is approved for implementation, a writing group will be established consisting of the proposers (up to 3 persons that were centrally involved in the development of the concept) and members of the COBRA group (or other appointed cohort representatives). All persons involved in the process of reviewing these research concepts are bound by confidentiality. Medisch Ethische Toetsingscommissie, Academisch Medisch Centrum, Universiteit van Amsterdam Academisch Medisch Centrum, XT4-140 Meibergdreef 9, 1105 AZ Amsterdam, The Netherlands. UK National Research Ethics Service (NRES), Charing Cross Hospital, Fulham W6 8RF, London, UK.
