# Supplementary material for: Biomarkers of central and peripheral inflammation mediate the association between HIV and depressive symptoms
Source: Transl Psychiatry. 2023 Jun 6;13:190. doi: 10.1038/s41398-023-02489-0 (PMC10244452; doi:10.1038/s41398-023-02489-0)
Supplement: Supplementary file 1 — Supp File 1 - Undetectable Biomarkers [file 41398_2023_2489_MOESM1_ESM.docx]

**Supplementary Table 1**. Number of participants with biomarker value set to a value of half the detection limit, in the full sample and stratified by HIV status.

|  | **n with data** | **n at a detection limit, overall** | **n at a detection limit, with HIV** | **n at a detection limit, without HIV** |
| --- | --- | --- | --- | --- |
| *Soluble biomarkers measured in all participants, where possible* | | | | |
| ***Plasma*** | | | | |
| CRP | 204 | 0 | 0 | 0 |
| I-FABP | 202 | 0 | 0 | 0 |
| Kyn:Trp | 203 | 0 | 0 | 0 |
| Neopterin | 203 | 0 | 0 | 0 |
| NFL | 202 | 0 | 0 | 0 |
| sCD14 | 201 | 0 | 0 | 0 |
| sCD16 | 202 | 0 | 0 | 0 |
| sCD163 | 204 | 0 | 0 | 0 |
| ***CSF*** | | | | |
| Kyn:Trp | 202 | 86 | 43 | 43 |
| Neopterin | 202 | 0 | 0 | 0 |
| NFL | 203 | 0 | 0 | 0 |
| sCD14 | 202 | 6 | 4 | 2 |
| sCD163 | 203 | 12 | 11 | 1 |
| *Soluble biomarkers measured in a subset of 78 participants* | | | | |
| ***Plasma*** | | | | |
| IL-6 | 78 | 26 | 14 | 12 |
| IP-10 / CXCL10 | 78 | 0 | 0 | 0 |
| MCP-1 / CCL2 | 78 | 0 | 0 | 0 |
| MIG / CXCL9 | 78 | 31 | 17 | 14 |
| MIP1-α / CCL3 | 78 | 67 | 34 | 33 |
| RANTES / CCL5 | 78 | 0 | 0 | 0 |
| TNF-α | 78 | 18 | 4 | 14 |
| ***CSF*** | | | | |
| IL-6 | 78 | 15 | 9 | 6 |
| IP-10 / CXCL10 | 78 | 0 | 0 | 0 |
| MCP-1 / CCL2 | 78 | 0 | 0 | 0 |
| MIG / CXCL9 | 78 | 29 | 17 | 12 |
| MIP1-α / CCL3 | 78 | 38 | 22 | 16 |
| RANTES / CCL5 | 78 | 45 | 24 | 21 |
| TNF-α | 78 | 42 | 19 | 23 |
