# Supplementary material for: Biomarkers of central and peripheral inflammation mediate the association between HIV and depressive symptoms
Source: Transl Psychiatry. 2023 Jun 6;13:190. doi: 10.1038/s41398-023-02489-0 (PMC10244452; doi:10.1038/s41398-023-02489-0)
Supplement: Supplementary file 2 — Supp File 2 - Statistical Analyses [file 41398_2023_2489_MOESM2_ESM.docx]

# **Statistical Analysis Methods**

- We summarised baseline variables using counts (proportions) for categorical variables and medians (interquartile ranges (IQRs)) for continuous outcomes. Univariate comparisons by HIV status were conducted using Wilcoxon-rank sum, Chi-squared (with Yates correction), Cochran-Armitage and Fisher’s exact tests, as appropriate.
- There were missing data in a few of the variables in which plausible values were assumed prior to summarising/analysing. Specifically, there were 2 (1 HIV-positive and 1 HIV-negative) participants missing data on ethnicity that were from the Amsterdam site and assumed to be white, given most participants from this site were white. Similarly, there was 1 HIV-negative participant missing data on current alcohol use that was assumed to be a current user given most participants were current users at baseline, and there was 1 HIV-negative participant missing data on ever injected drugs that was assumed to have never injected drugs given most participants never injected drugs at baseline.
- The PHQ-9 score was summarised as a continuous variable and a dichotomous variable (PHQ-9 score > 4 classified as ‘Any Depressive Symptoms’, primary outcome).
- We summarised biomarker variables collected at the baseline COBRA visit using medians (IQRs) by HIV status and overall (as well as by the dichotomised outcome). Due to high costs, plasma cytokines were only measured in a subset of 78. These 78 participants were randomly selected with equal numbers across COBRA age groups (45–50 years, 51–55 years, 56–60 years, 61–65 years, 66–70 years), except for the oldest age group (>70 years) where few individuals were available. All other biomarkers used in these analyses were measured in all participants, where possible.
- We also fitted separate linear regression models for each biomarker using the log_2_ transformed biomarker concentration as the outcome. These analyses were included to assess the associations between HIV status and each biomarker.
- **Primary Analyses:** We used logistic regression to first explore the main effect of HIV status on the presence of ‘Any Depressive Symptoms’ (PHQ-9 score > 4). We first adjusted the model for sociodemographic factors: age (continuous), sex, ethnicity, and years of education (continuous). We then sequentially adjusted the model for each (log_2_ transformed) biomarker separately to determine whether any of the biomarkers appeared to mediate the relationship between HIV status and ‘Any Depressive Symptoms’. Logistic regression models that included neurometabolites were also adjusted for MRI scanner in all models (1 scanner was used at the London site, and 2 scanners were used at the Amsterdam site). We report the parameter estimate for HIV status for all models. Additionally, we report profile-likelihood confidence intervals due to the small number of events in the outcome.
- Adjustment for biomarkers individually that yielded a > 10% reduction in the HIV status odds ratio (OR) were considered likely mediators of the association between HIV status and ‘Any Depressive Symptoms’. A reduction rather than an increase in the effect estimate after adjustment is commonly used to identify potential mediator variables.
- **Sensitivity Analyses:** Linear regression was used for sensitivity analyses, using the PHQ-9 score as a (continuous) outcome. The same sequential adjustments were made as noted above for the primary logistic regression analysis.
- All analyses were conducted using listwise deletion, only including participants with data available on all variables used in an analysis. Analyses were performed using R version 4.1.0, with two-sided p-values <0.05 considered to be statistically significant.
