# Supplementary material for: Biomarkers of central and peripheral inflammation mediate the association between HIV and depressive symptoms
Source: Transl Psychiatry. 2023 Jun 6;13:190. doi: 10.1038/s41398-023-02489-0 (PMC10244452; doi:10.1038/s41398-023-02489-0)
Supplement: Supplementary file 4 — Supp File 4 - Biomarkers by HIV Status (Linear Regression) [file 41398_2023_2489_MOESM4_ESM.docx]

| **Supplementary Table 2.** Estimated Mean Difference (EMD, with 95% confidence interval [CI]) for the association between HIV status and each log_2_-transformed biomarker separately, before and after adjustment for sociodemographic factors. | | | | | |  |
| --- | --- | --- | --- | --- | --- | --- |
| **Biomarker** | ***N*** | **Estimated Mean Difference (95% CI)** | | | |  |
|  |  | **Unadjusted** | ***p* value** | **Adjusted for age, sex, ethnicity, years of education** | ***p* value** |  |
| **Neurometabolites*** | | | | | | |
| *Myo*-inositol | | | | | |  |
| FWM | 161 | 0.98 (0.89, 1.07) | 0.63 | 0.98 (0.89, 1.07) | 0.64 |  |
| Putamen | 63 | 1.07 (0.90, 1.27) | 0.46 |  |  |  |
| Choline-containing compounds | | | | | |  |
| FWM | 184 | 0.97 (0.92, 1.01) | 0.17 | 0.97 (0.93, 1.02) | 0.28 |  |
| Putamen | 128 | 0.95 (0.86, 1.05) | 0.33 | 0.95 (0.86, 1.05) | 0.29 |  |
|  |  |  | |  | |  |
| **Circulating biomarkers**  *measured in all participants, where possible* | | | | | | |
| **Plasma** | | | | | | |
| CRP | 204 | 1.67 (1.21, 2.32) | 0.002 | 1.64 (1.18, 2.29) | 0.004 |  |
| I-FABP | 202 | 1.79 (1.50, 2.13) | <0.001 | 1.78 (1.49, 2.13) | <0.001 |  |
| Kyn:Trp | 203 | 1.12 (1.03, 1.22) | 0.01 | 1.14 (1.05, 1.24) | 0.002 |  |
| Neopterin | 203 | 1.32 (1.17, 1.49) | <0.001 | 1.38 (1.23, 1.55) | <0.001 |  |
| NFL | 202 | 1.09 (0.96, 1.25) | 0.19 | 1.15 (1.03, 1.29) | 0.01 |  |
| sCD14 | 201 | 1.16 (1.05, 1.27) | 0.002 | 1.19 (1.08, 1.30) | <0.001 |  |
| sCD16 | 202 | 1.13 (0.97, 1.32) | 0.12 | 1.18 (1.00, 1.38) | 0.04 |  |
| sCD163 | 204 | 1.20 (1.05, 1.38) | 0.01 | 1.20 (1.04, 1.37) | 0.01 |  |
| **CSF** | | | | | | |
| Kyn:Trp | 202 | 1.43 (1.16, 1.76) | 0.001 | 1.42 (1.15, 1.75) | 0.001 |  |
| Neopterin | 202 | 1.15 (1.04, 1.28) | 0.01 | 1.17 (1.06, 1.30) | 0.003 |  |
| NFL | 203 | 1.05 (0.92, 1.21) | 0.45 | 1.10 (0.99, 1.23) | 0.08 |  |
| sCD14 | 202 | 0.59 (0.37, 0.96) | 0.03 | 0.61 (0.37, 0.99) | 0.05 |  |
| sCD163 | 203 | 0.69 (0.48, 0.98) | 0.04 | 0.71 (0.50, 1.02) | 0.06 |  |
|  |  |  | |  | |  |
| **Circulating biomarkers**  *measured in a subset of 78 participants* | | | | | | |
| **Plasma** | | | | | | |
| IL-6 | 78 | 0.62 (0.21, 1.83) | 0.38 | 0.61 (0.21, 1.79) | 0.36 |  |
| IP-10 / CXCL10 | 78 | 1.21 (0.92, 1.61) | 0.17 | 1.15 (0.87, 1.51) | 0.32 |  |
| MCP-1 / CCL2 | 78 | 0.94 (0.77, 1.14) | 0.52 | 1.01 (0.83, 1.23) | 0.90 |  |
| MIG / CXCL9 | 78 | 0.82 (0.61, 1.11) | 0.20 | 0.85 (0.62, 1.16) | 0.30 |  |
| MIP1α / CCL3 | 78 | 0.91 (0.65, 1.29) | 0.60 | 0.90 (0.63, 1.29) | 0.56 |  |
| RANTES / CCL5 | 78 | 0.76 (0.48, 1.22) | 0.25 | 0.78 (0.48, 1.29) | 0.33 |  |
| TNF-α | 78 | 3.05 (1.20, 7.77) | 0.02 | 3.51 (1.33, 9.30) | 0.01 |  |
| **CSF** | | | | | | |
| IL-6 | 78 | 0.68 (0.25, 1.85) | 0.44 | 0.80 (0.30, 2.09) | 0.64 |  |
| IP-10 / CXCL10 | 78 | 1.39 (1.07, 1.81) | 0.02 | 1.35 (1.02, 1.78) | 0.04 |  |
| MCP-1 / CCL2 | 78 | 1.01 (0.87, 1.18) | 0.90 | 1.04 (0.89, 1.21) | 0.62 |  |
| MIG / CXCL9 | 78 | 0.93 (0.65, 1.34) | 0.70 | 0.94 (0.64, 1.36) | 0.73 |  |
| MIP1α / CCL3 | 78 | 0.70 (0.43, 1.15) | 0.15 | 0.72 (0.43, 1.22) | 0.22 |  |
| RANTES / CCL5 | 78 | 0.47 (0.10, 2.16) | 0.33 | 0.84 (0.18, 3.91) | 0.82 |  |
| TNF-α | 78 | 1.46 (0.56, 3.80) | 0.44 | 1.43 (0.53, 3.87) | 0.47 |  |
|  |  |  | |  | |  |

* All models which included neurometabolite measures were corrected for scanner.
