# Supplementary material for: Biomarkers of central and peripheral inflammation mediate the association between HIV and depressive symptoms
Source: Transl Psychiatry. 2023 Jun 6;13:190. doi: 10.1038/s41398-023-02489-0 (PMC10244452; doi:10.1038/s41398-023-02489-0)
Supplement: Supplementary file 5 — Supp File 5 - Sensitivity Analyses (Linear Regression) [file 41398_2023_2489_MOESM5_ESM.docx]

| **Supplementary Table 3: Sensitivity Analyses.** Estimated Mean Difference (EMD, with 95% confidence interval [CI]) for the association between HIV status and PHQ-9 score, before and after adjustment for each biomarker fitted separately, and percentage change in EMD from each model. | | | | |
| --- | --- | --- | --- | --- |
| **Biomarker** | ***N*** | **Estimated Mean Difference (95% CI)** Adjusted for HIV status and sociodemographic factors | | **% Change in EMD** |
|  |  | **Before adjusting for biomarker** | **Adjusted for biomarker** |  |
| **Neurometabolites*** | | | | |
| *Myo*-inositol | | | |  |
| FWM | 161 | 1.40 (0.25, 2.54) | 1.52 (0.36, 2.69) | 8.6 |
| Putamen | 63 | 3.08 (0.59, 5.56) | 1.53 (0.46, 2.60) | 4.5 |
| Choline-containing compounds | | | | |
| FWM | 184 | 1.42 (0.36, 2.47) | 2.91 (1.21, 7.74) | 7.7 |
| Putamen | 128 | 2.52 (1.14, 3.89) | 2.70 (1.30, 4.10) | 7.1 |
|  |  |  |  |  |
| **Circulating biomarkers**  *measured in all participants, where possible* | | | | |
| **Plasma** | | | | |
| CRP | 204 | 1.75 (0.73, 2.78) | 1.53 (0.49, 2.57) | -12.6 |
| I-FABP | 202 | 1.82 (0.79, 2.85) | 1.81 (0.68, 2.94) | -0.5 |
| Kyn:Trp | 203 | 1.80 (0.77, 2.83) | 1.60 (0.55, 2.65) | -11.1 |
| Neopterin | 203 | 1.80 (0.77, 2.83) | 1.66 (0.56, 2.77) | -7.8 |
| NFL | 202 | 1.62 (0.62, 2.62) | 1.47 (0.46, 2.48) | -9.3 |
| sCD14 | 201 | 1.72 (0.69, 2.76) | 1.88 (0.81, 2.95) | 9.3 |
| sCD16 | 202 | 1.79 (0.76, 2.82) | 1.65 (0.61, 2.68) | -7.8 |
| sCD163 | 204 | 1.75 (0.73, 2.78) | 1.79 (0.74, 2.83) | 2.3 |
| **CSF** | | | | |
| Kyn:Trp | 202 | 1.57 (0.57, 2.57) | 1.63 (0.60, 2.66) | 3.8 |
| Neopterin | 202 | 1.57 (0.57, 2.57) | 1.51 (0.48, 2.53) | -3.8 |
| NFL | 203 | 1.60 (0.60, 2.60) | 1.63 (0.62, 2.63) | 1.9 |
| sCD14 | 202 | 1.58 (0.57, 2.58) | 1.65 (0.64, 2.66) | 4.4 |
| sCD163 | 203 | 1.60 (0.60, 2.60) | 1.69 (0.68, 2.69) | 5.6 |
|  |  |  |  |  |
| **Circulating biomarkers**  *measured in a subset of 78 participants* | | | | |
| **Plasma** | | | | |
| IL-6 | 78 | 1.24 (-0.53, 3.02) | 1.17 (-0.63, 2.96) | -5.6 |
| IP-10 / CXCL10 | 78 | 1.24 (-0.53, 3.02) | 1.28 (-0.52, 3.08) | 3.2 |
| MCP-1 / CCL2 | 78 | 1.24 (-0.53, 3.02) | 1.24 (-0.55, 3.03) | 0.0 |
| MIG / CXCL9 | 78 | 1.24 (-0.53, 3.02) | 1.15 (-0.64, 2.95) | -7.3 |
| MIP1α / CCL3 | 78 | 1.24 (-0.53, 3.02) | 1.29 (-0.49, 3.08) | 4.0 |
| RANTES / CCL5 | 78 | 1.24 (-0.53, 3.02) | 1.21 (-0.59, 3.01) | -2.4 |
| TNF-α | 78 | 1.24 (-0.53, 3.02) | 0.87 (-0.97, 2.72) | -29.8 |
| **CSF** | | | | |
| IL-6 | 78 | 1.24 (-0.53, 3.02) | 1.15 (-0.60, 2.90) | -7.3 |
| IP-10 / CXCL10 | 78 | 1.24 (-0.53, 3.02) | 1.16 (-0.68, 3.01) | -6.5 |
| MCP-1 / CCL2 | 78 | 1.24 (-0.53, 3.02) | 1.27 (-0.52, 3.06) | 2.4 |
| MIG / CXCL9 | 78 | 1.24 (-0.53, 3.02) | 1.22 (-0.57, 3.00) | -1.6 |
| MIP1α / CCL3 | 78 | 1.24 (-0.53, 3.02) | 1.01 (-0.76, 2.78) | -18.5 |
| RANTES / CCL5 | 78 | 1.24 (-0.53, 3.02) | 1.23 (-0.55, 3.02) | -0.8 |
| TNF-α | 78 | 1.24 (-0.53, 3.02) | 1.22 (-0.57, 3.02) | -1.6 |
|  |  |  |  |  |

* Models which included neurometabolite measures were further corrected for scanner.
