# Supplementary figures and images for: Biomarkers of central and peripheral inflammation mediate the association between HIV and depressive symptoms
Source: Transl Psychiatry. 2023 Jun 6;13:190. doi: 10.1038/s41398-023-02489-0 (PMC10244452; doi:10.1038/s41398-023-02489-0)

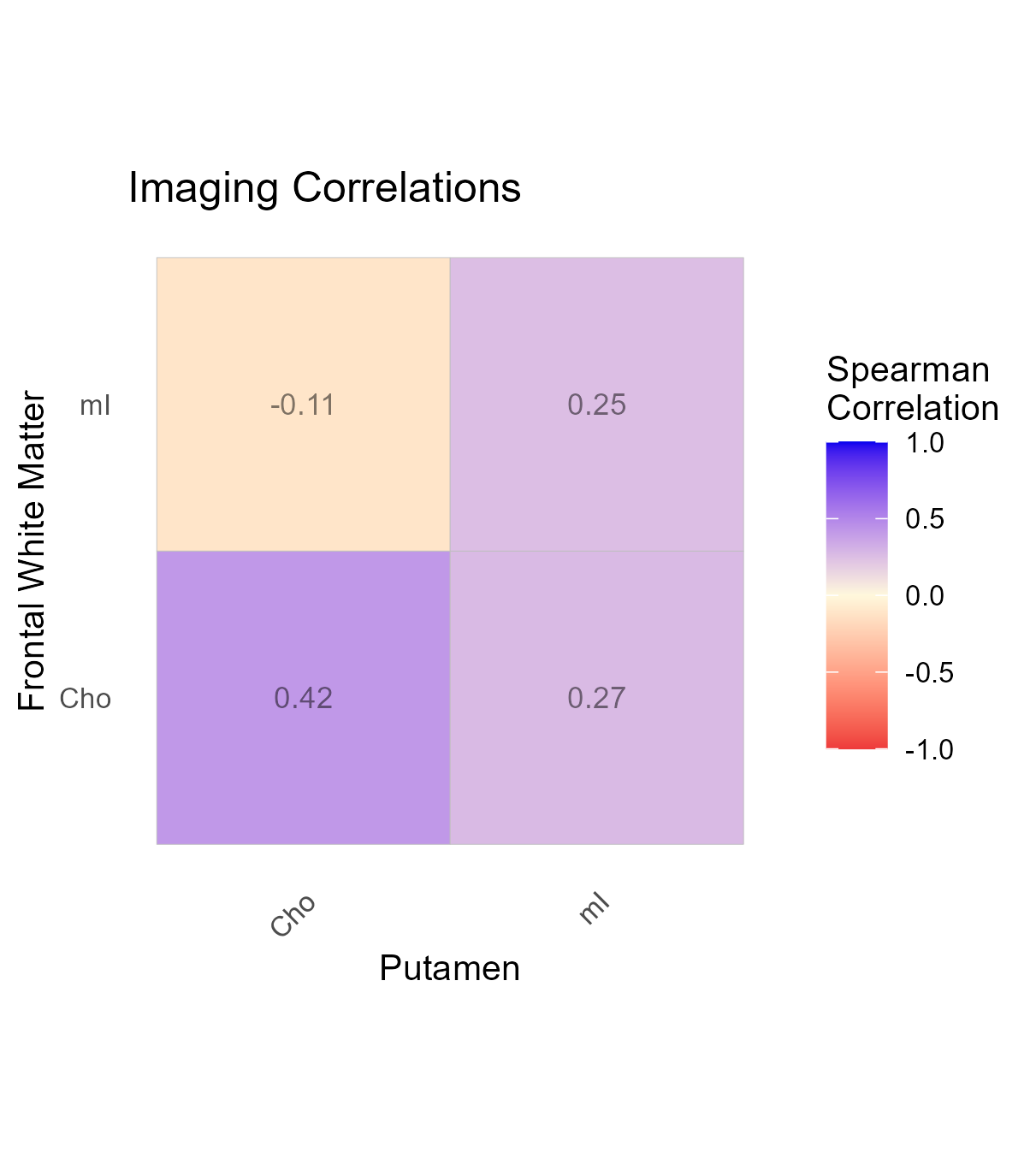

Supplement: Supplementary file 6 — Supp Figure 1 - Imaging Correlations [file 41398_2023_2489_MOESM6_ESM.png]

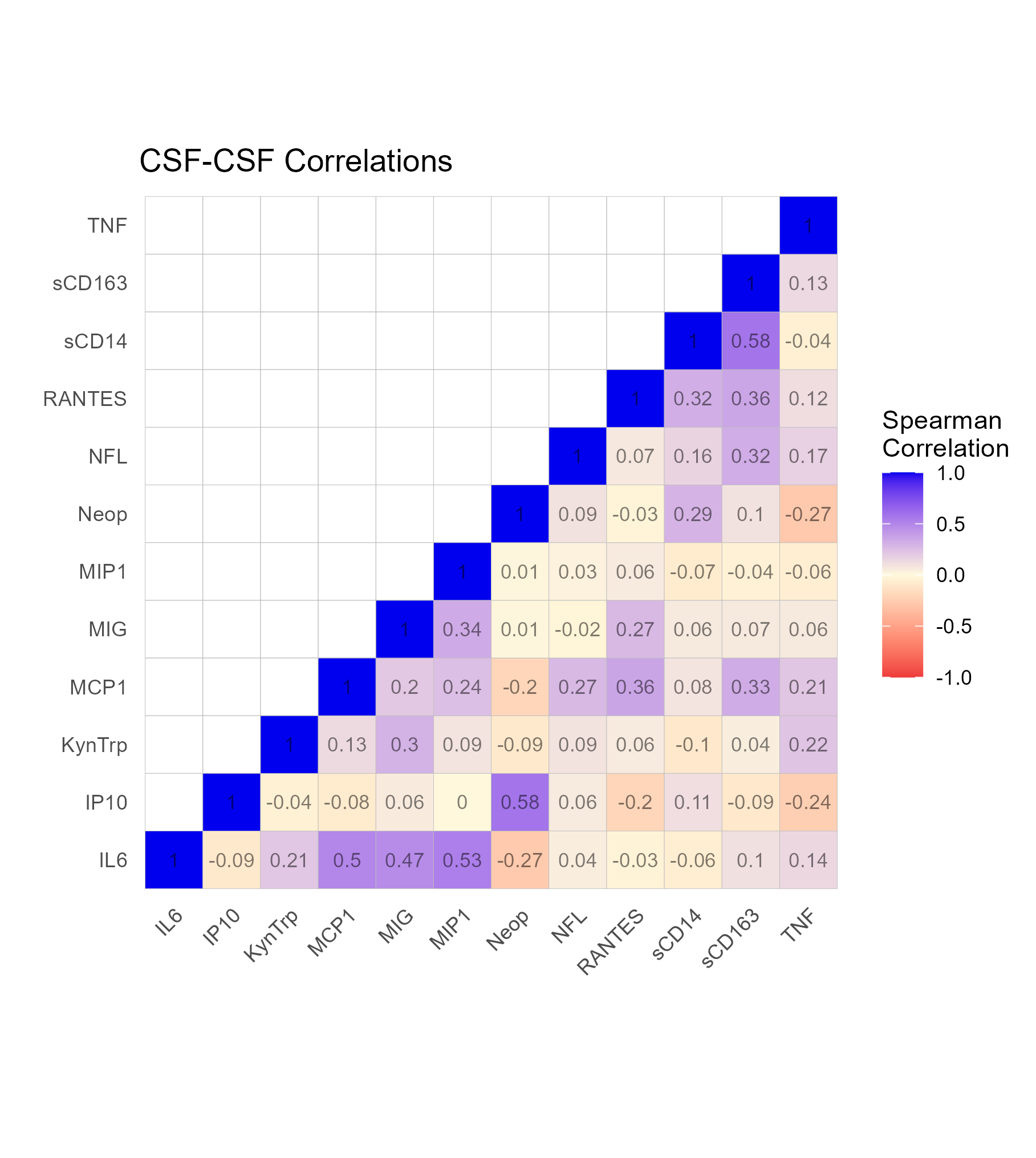

Supplement: Supplementary file 7 — Supp Figure 2 - CSF-CSF Correlations [file 41398_2023_2489_MOESM7_ESM.png]

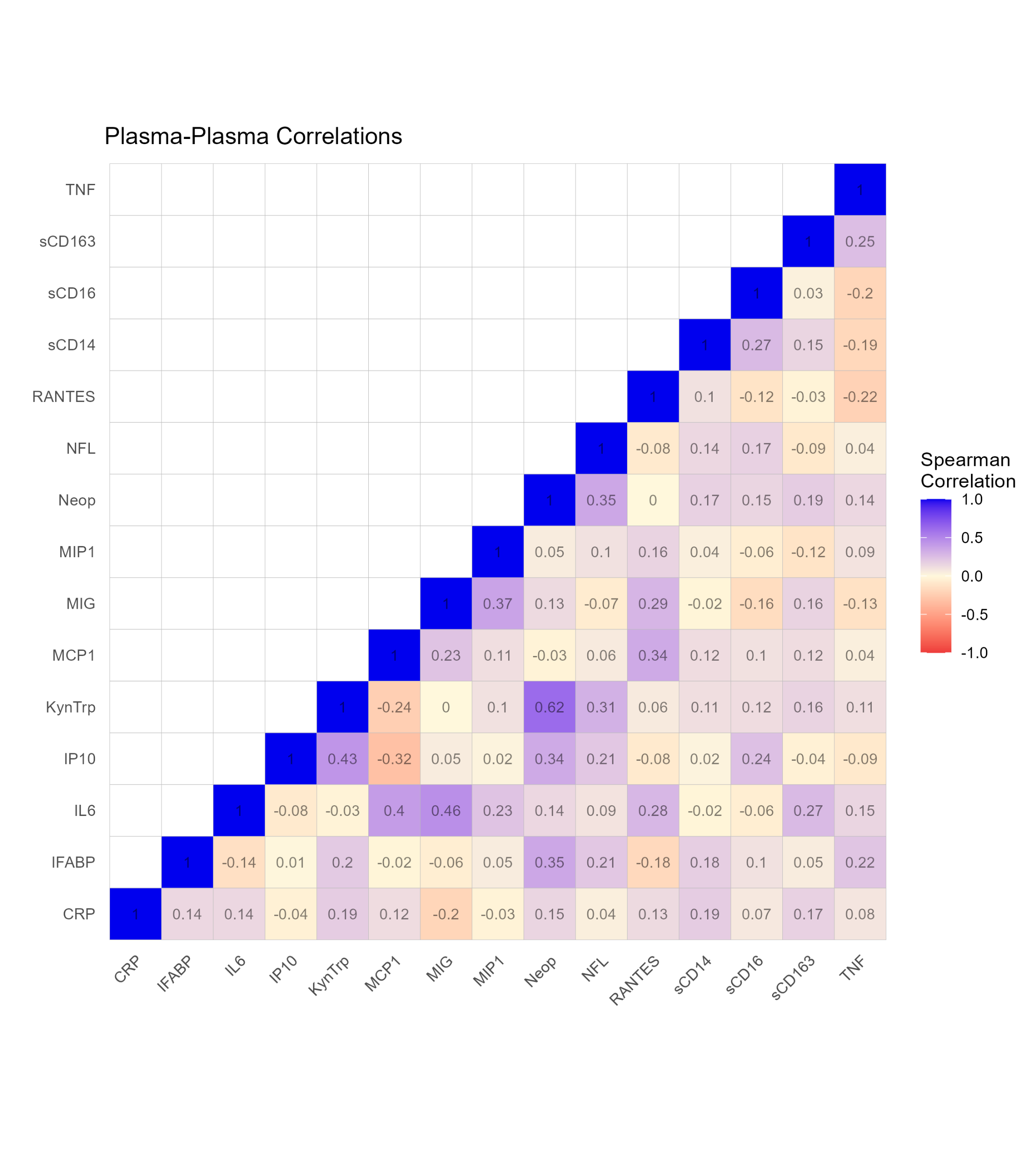

Supplement: Supplementary file 8 — Supp Figure 3 - Plasma-Plasma Correlations [file 41398_2023_2489_MOESM8_ESM.png]
