# Supplementary material for: Biomarkers of central and peripheral inflammation mediate the association between HIV and depressive symptoms
Source: Transl Psychiatry. 2023 Jun 6;13:190. doi: 10.1038/s41398-023-02489-0 (PMC10244452; doi:10.1038/s41398-023-02489-0)
Supplement: Supplementary file 9 — Supplementary Figures [file 41398_2023_2489_MOESM9_ESM.docx]

**Supplementary Figure 1. Correlations between frontal white matter (FWM) and putamen measurements of neurometabolites measured in the current study.** Strength of correlations are graded across a 3-point scale: -1.0 (in deep red), 0.0 (in light yellow), and +1.0 (in deep blue).

**Supplementary Figure 2. Correlations between cerebrospinal fluid (CSF) measurements of soluble biomarkers measured in the current study.** Strength of correlations are graded across a 3-point scale: -1.0 (in deep red), 0.0 (in light yellow), and +1.0 (in deep blue).

**Supplementary Figure 3. Correlations between cerebrospinal fluid (CSF) measurements of soluble biomarkers measured in the current study.** Strength of correlations are graded across a 3-point scale: -1.0 (in deep red), 0.0 (in light yellow), and +1.0 (in deep blue).
